# Supplementary material for: Assessment of Volatiles and Polyphenol Content, Physicochemical Parameters and Antioxidant Activity in Beers with Dotted Hawthorn (Crataegus punctata)
Source: Foods. 2020 Jun 11;9(6):775. doi: 10.3390/foods9060775 (PMC7353495; doi:10.3390/foods9060775)

Table S1. Content of volatile compounds in hawthorn fruit and juice

	Compound name	Retention time	Chemical family	Kovats Indices		CAS Number	Compounds in hawthorn	
				KI exp.	KI NIST		Juice (µg / 100 mL	Fruit (µg / 100 g)
1	Butanoic acid, ethyl ester	5.852	Esters	799	802	105-54-4	0.6795 ± 0.3134	5,3896 ± 0,7949
2	Butanoic acid, 2- methyl-, ethyl ester	7.261	Esters	850	849	7452-79-1	1.0285 ± 0.6492	4,4532 ± 0,6767
3	1-Hexanol	7.857	Alcohols	873	868	111-27-3	0.1524 ± 0.0636	0,9628 ± 0,3095
4	Hexanoic acid, methyl ester	11.656	Esters	928	925	110-93-0	1.7633 ± 0.6562	1,5879 ± 0,7162
5	β-Myrcene	11.782	Monoterpenes	994	991	123-35-3	0.2287 ± 0.1536	1,7479 ± 0,3877
6	Butanoic acid, butyl ester	11.942	Esters	999	995	109-21-7	0.3394 ± 0.2478	0,3808 ± 0,2584
8	Hexanoic acid, ethyl ester	12.034	Esters	1001	1000	123-66-0	3.8006 ± 1.0216	124,1183 ± 19,4641
9	Acetic acid, hexyl ester	12.508	Esters	1017	1011	123-35-3	0.0224 ± 0.0106	1,4218 ± 0,4809
10	Propanoic acid, 2-methyl-, 2-methylbutyl ester	12.597	Esters	1020	1016	2445-69-4	0.0094 ± 0.0076	6,53292 ± 1,9561
11	p-Cymene	12.638	Aromatic hydrocarbons	1027	1025	99-87-6	0.0853 ± 0.0442	7,7414 ± 1,8577
12	Limonene	12.891	Monoterpenes	1031	1030	5989-54-8	0.3124 ± 0.1056	2,5159 ± 0,8843
13	Butanoic acid, 3-methylbutyl ester	13.678	Esters	1060	1056	106-27-4	trace	0,9028 ± 0,2771
14	Ethyl 5-methylhexanoate	14.127	Esters	1067	1072	10236-10-9	0.1392 ± 0.0651	0,9494 ± 0,2867
15	1-Octanol	14.363	Alcohols	1075	1071	111-87-5	Trace	66,5846 ± 13,09
16	2-Nonanone	14.891	Ketones	1095	1092	821-55-6	Trace	453,5589 ± 66,58
17	Linalool	15.234	Alcohols	1100	1099	78-70-6	11.0347 ± 3.6252	7,9473 ± 1,1866
18	2-Nonen-1-ol	15.311	Alcohols	1102	1105	22104-79-6	0.2874 ± 0.0833	1,4866 ± 0,3798
19	Phenylethyl Alcohol	15.564	Esters	1113	1116	1960-12-08	38.1292 ± 11.0504	5,0293 ± 1,3652
20	Octanoic acid, methyl ester	15.945	Esters	1127	1126	106-32-1	0.4435 ± 0.1268	3,9236 ± 1,3589
21	Endo-Borneol*	17.143	Monoterpenes	1175	1167	464-43-7	trace	1,8397 ± 0,2957
22	Benzoic acid, ethyl ester	17.332	Esters	1181	1171	93-89-0	1.0604 ± 0.2148	3,0349 ± 1,157
23	Octanoic acid	17.685	Organic acids	1193	1180	124-07-2	26.5991 ± 8.6384	3,6881 ± 0,3187
24	α-Terpineol	17.753	Monoterpene	1193	1190	98-55-5	71.0423 ± 16.9588	1025,4730 ± 146,5551
25	Octanoic acid, ethyl ester	17.796	Esters	1195	1196	106-32-1	77.0187 ± 15.9404	0,7109 ± 0,2062
26	Decanal	18.053	Aldehydes	1209	1206	112-31-2	1.4357 ± 0.5831	15,4079 ± 2,6231
27	Acetic acid, octyl ester	18.163	Esters	1218	1211	112-14-1	trace	46,4634 ± 6,5099
28	Citronellol	18.46	Alcohols	1238	1229	106-22-9	4.4067 ± 1.2383	trace
29	Benzeneacetic acid, ethyl ester	18.683	Esters	1253	1246	101-97-3	0.9544 ± 0.3578	66,5008 ± 11,0401
30	Isopentyl hexanoate	18.775	Esters	1255	1252	2198-61-0	0.0694 ± 0.0352	0,2594 ± 0,0963
31	Acetic acid, 2-phenylethyl ester	18.912	Esters	1264	1258	103-45-7	31.0218 ± 6.4922	3,7554 ± 0,9187
32	1-Decanol	19.086	Alcohols	1277	1273	112-31-2	6.1448 ± 1.9622	0,1118 ± 0,0376
33	2,4-Heptadienoic acid, 6-methyl-, ethyl ester	19.223	Esters	1284	1293	10236-06-03	1.5659 ± 0.8291	0,8776 ± 0,2989

34	Methyl geranate	19.624	Esters	1338	1326	214-712-6	0.9231 ± 0.3622	14,2427 ± 2,6893
35	Octanoic acid, 2-methylpropyl ester	19.951	Esters	1352	1348	5461-06-03	0.6129 ± 0.0908	1,3142 ± 0,3884
36	Citronellol acetate	20.044	Esters	1358	1354	150-84-5	0.0889 ± 0.0542	1,2514 ± 0,6041
37	Unknown sesquiterpene	20.149	Sesquiterpenes	1369			3.6706 ± 0.9262	0,2468 ± 0,0589
38	Ethyl (4E)-4-decenoate	20.286	Esters	1384	1377	76649-16-6	0.2617 ± 0.0828	5,5417 ± 1,2349
39	Ethyl trans-2-decenoate	20.373	Esters	1390	1389	7367-88-6	2.9772 ± 1.0432	86,5502 ± 12,5693
40	Decanoic acid, ethyl ester	20.456	Esters	1397	1396	110-38-3	36.3976 ± 9.2634	10,1857 ± 2,0047
41	Dodecanal	20.561	Aldehydes	1412	1409	112-54-9	Trace	1,8214 ± 0,7885
42	β-Caryophyllene	20.763	Sesquiterpenes	1438	1419	87-44-5	0.1748 ± 0.0818	4,6222 ± 0,8542
43	Octanoic acid, 3-methylbutyl ester	20.891	Esters	1454	1446	2035-99-6	1.0078 ± 0.8784	14,6308 ± 2,9207
44	(E)-β-Famesene	20.967	Sesquiterpenes	1463	1457	18794-84-8	0.6222 ± 0.2948	21,0358 ± 6,3506
45	Humulene	21.058	Sesquiterpenes	1473	1454	6753-98-6	0.4218 ± 0.3456	13,3957 ± 4,9045
46	α-Muurolene	21.233	Sesquiterpenes	1494	1485	10208-80-7	Trace	1,3857 ± 0,3163
47	Pentadecane	21.383	Hydrocarbons	1502	1500	629-62-9	Trace	2,3318 ± 0,6294
48	δ-Cadinene	21.482	Sesquiterpenes	1513	1524	483-76-1	0.1554 ± 0.0876	5,4305 ± 1,3873
49	Unknown compound	21.687		1557			0.4328 ± 0.2566	0,0068 ± 0,0046
50	β-Calacorene	21.739	Sesquiterpenes	1565	1563	50277-34-4	0.3988 ± 0.1474	0,4826 ± 0,2881
51	Nerolidol	21.774	Sesquiterpenes	1572	1565	7212-44-4	0.1008 ± 0.0478	3,3659 ± 0,8891
52	Dodecanoic acid, ethyl ester	21.993	Esters	1592	1595	106-33-2	3.1354 ± 0.9824	161,3785 ± 55,4602
53	Humulene epoxide I	22.183	Sesquiterpenes	1609	1604	19888-33-6	0.2229 ± 0.1474	25,4797 ± 6,5237
	Total						331.3798	2240,0588

Figure S1. Mass spectrum of unknown sesquiterpene

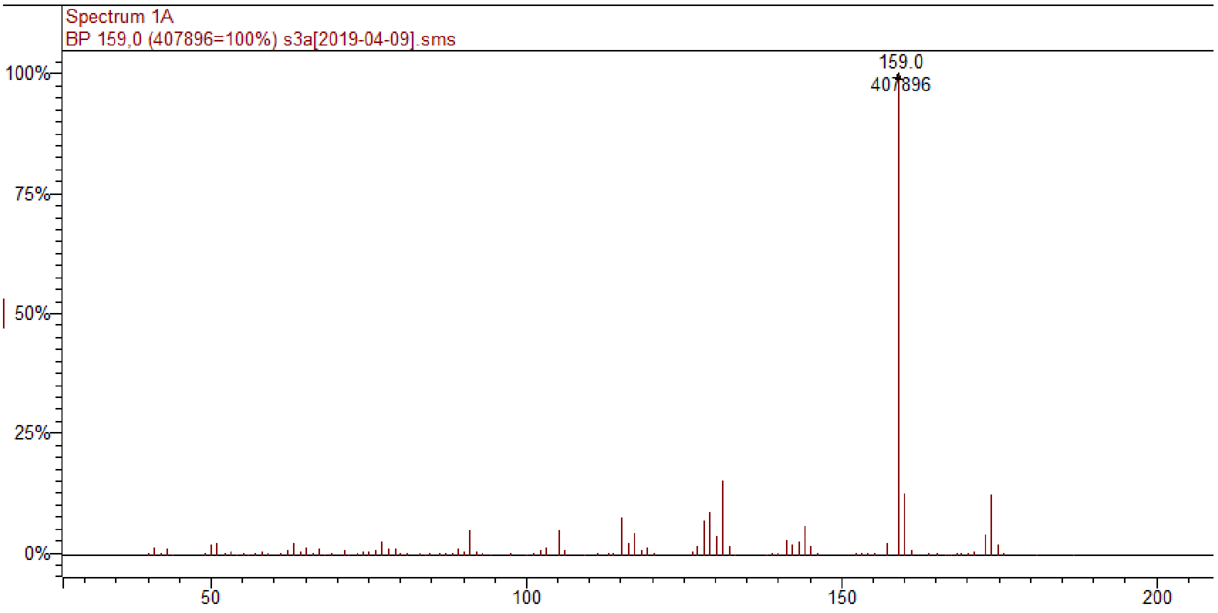

Figure S2. Mass spectrum of unknown compound

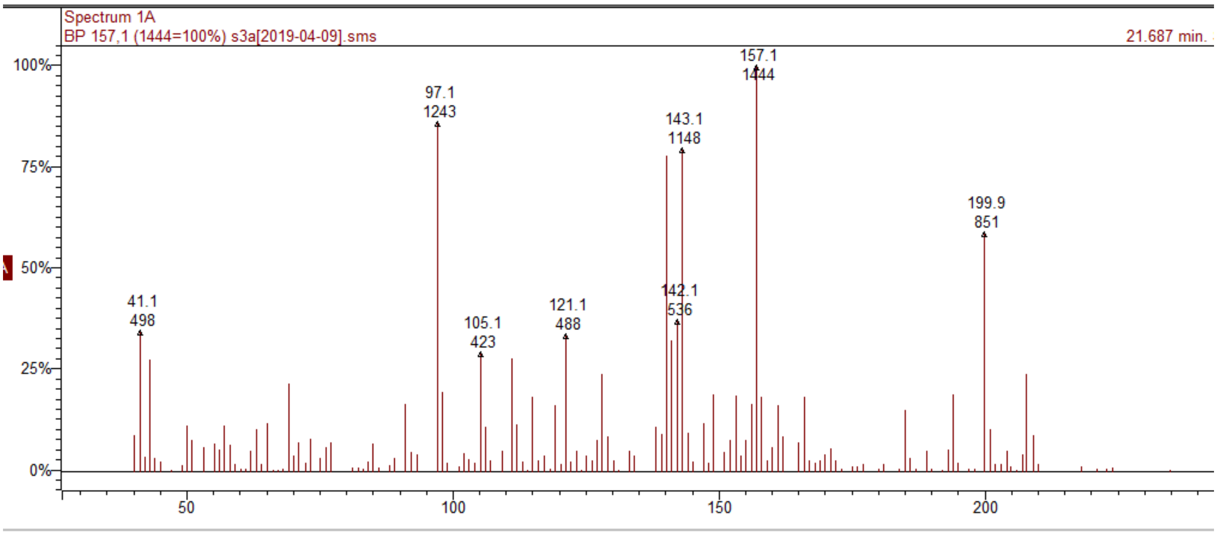

Supplement: Supplementary file 1 [file foods-09-00775-s001.pdf]
